# Genome sequencing of the NIES Cyanobacteria collection with a focus on the heterocyst-forming clade

**DOI:** 10.1093/dnares/dsab024

**Published:** 2021-10-22

**Authors:** Yuu Hirose, Yoshiyuki Ohtsubo, Naomi Misawa, Chinatsu Yonekawa, Nobuyoshi Nagao, Yohei Shimura, Takatomo Fujisawa, Yu Kanesaki, Hiroshi Katoh, Mitsunori Katayama, Haruyo Yamaguchi, Hirofumi Yoshikawa, Masahiko Ikeuchi, Toshihiko Eki, Yasukazu Nakamura, Masanobu Kawachi

**Affiliations:** 1 Department of Applied Chemistry and Life Science, Toyohashi University of Technology, 1-1 Hibarigaoka, Tenpaku, Toyohashi, Aichi, 441-8580, Japan; 2 Graduate School of Life Sciences, Tohoku University, 2-1-1 Katahira, Aoba, Sendai, Miyagi, 980-0812, Japan; 3 Biodiversity Division, National Institute for Environmental Studies, 16-1 Onogawa, Tsukuba, Ibaraki, 305-8506, Japan; 4 Department of Informatics, National Institute of Genetics, 1111 Yata, Mishima, Shizuoka, 411-8540, Japan; 5 Research Institute of Green Science and Technology, Shizuoka University, 836 Oya, Suruga, Shizuoka, Shizuoka, 422-8529, Japan; 6 Advanced Science Research Promotion Center, Mie University, 1577 Kurima, Tsu, Mie, 514-8507, Japan; 7 College of Industrial Technology, Nihon University, 1-2-1 Izumi, Narashino, Chiba, 275-8575, Japan; 8 Department of Bioscience, Tokyo University of Agriculture, 1-1-1 Sakuragaoka, Setagaya, Tokyo, 156-8502, Japan; 9 Department of Life Sciences (Biology), The University of Tokyo, 3-8-1 Komaba, Meguro, Tokyo, Japan

**Keywords:** Cyanobacteria, genome, heterocyst, culture collection, taxonomy

## Abstract

Cyanobacteria are a diverse group of Gram-negative prokaryotes that perform oxygenic photosynthesis. Cyanobacteria have been used for research on photosynthesis and have attracted attention as a platform for biomaterial/biofuel production. Cyanobacteria are also present in almost all habitats on Earth and have extensive impacts on global ecosystems. Given their biological, economical, and ecological importance, the number of high-quality genome sequences for Cyanobacteria strains is limited. Here, we performed genome sequencing of Cyanobacteria strains in the National Institute for Environmental Studies microbial culture collection in Japan. We sequenced 28 strains that can form a heterocyst, a morphologically distinct cell that is specialized for fixing nitrogen, and 3 non-heterocystous strains. Using Illumina sequencing of paired-end and mate-pair libraries with *in silico* finishing, we constructed highly contiguous assemblies. We determined the phylogenetic relationship of the sequenced genome assemblies and found potential difficulties in the classification of certain heterocystous clades based on morphological observation. We also revealed a bias on the sequenced strains by the phylogenetic analysis of the 16S rRNA gene including unsequenced strains. Genome sequencing of Cyanobacteria strains deposited in worldwide culture collections will contribute to understanding the enormous genetic and phenotypic diversity within the phylum Cyanobacteria.

## 1. Introduction

Cyanobacteria (blue-green algae) are a diverse group of Gram-negative prokaryotes that perform oxygenic photosynthesis.^1^ Their photosynthetic activity transformed the ancient Earth’s atmosphere from anoxygenic to oxygenic conditions, which is considered to have occurred between 2.4 and 2.1 billion years ago.^2^ Cyanobacteria evolved to become the ancient origin of the chloroplast in plants and eukaryotic algae via primary endosymbiosis,^3^ which is considered to have occurred 1.5 billion years ago,^4^ although a consensus has not yet been reached for the unity of the origin of chloroplast genes.^5^ In addition to their application in the study of photosynthesis, Cyanobacteria also represent a potential platform for the production of biomaterial/biofuel such as alcohols, diols, fatty acids, and organic acids by metabolic engineering.^6^ Cyanobacteria are present in almost all habitats on Earth and have extensive impacts on the global ecosystems through carbon^7^ and nitrogen^8^ fixation and through the production of various secondary metabolites.^9^ They can live in extreme environments such as oligotrophic oceans,^7^ deserts,^10^ glaciers,^11^ polar regions,^12^ and hot springs.^13^ Diverse Cyanobacteria strains have been isolated from various environments and deposited in culture collections worldwide.

With online database searches, we found 15 worldwide culture collections that maintain more than 100 publicly available Cyanobacteria strains (Supplementary Table S1). For example, The University of Helsinki Cyanobacteria Culture collection in Finland maintains 919 strains, which is the largest number in our online search. The microbial culture collection of the National Institute for Environmental Studies (NIES) in Japan maintains 827 strains, half of which comprise bloom-forming strains such as *Microcystis* and *Dolichospermum*, as the NIES has been studying water quality management in lakes and marshes in Japan since the 1970s. The Freshwater Algae Culture Collection at the Institute of Hydrobiology in China maintains 822 strains. The Pasteur Culture Collection (PCC) in France maintains 474 strains, which include phylogenetically diverse strains isolated from a wide variety of habitats.

As Cyanobacteria were previously thought to be members of the eukaryotic algae, the names of their taxa have traditionally been governed under the nomenclature of the Botanical Code, which differs from the Bacterial Code.^14^ There are several taxonomic schemes of Cyanobacteria. Based on morphological observation by microscopy, Cyanobacteria have conventionally been classified into five sections.^15^ Section I consists of unicellular cyanobacteria strains that reproduce by binary fission. Section II consists of unicellular Cyanobacteria that reproduce by multiple fission to form small daughter cells (baeocytes). Section III consists of filamentous Cyanobacteria that divide linearly in a single plane and exist only as vegetative cells. Section IV consists of filamentous Cyanobacteria that divide in a single plane and form heterocysts, which are highly specialized cells that fix nitrogen and have a thick cell wall to provide a microoxic environment in which to protect nitrogenase from oxygen inactivation.^16^ Section V consists of filamentous Cyanobacteria that form heterocysts and divide in multiple planes to form branched filaments. Phylogenetic analysis of marker genes (e.g. 16S rRNA) has revealed the polyphyletic distribution of Sections I−III strains, suggesting a loss and recovery of multicellularity during their evolution.^17^ Recently, taxonomic assignment of Cyanobacteria has been widely performed using the “polyphasic approach,” which combines phylogenetic data for marker gene(s), morphological data based on light, and electron microscopy, and ecophysiological data.^18^ Up-to-date taxonomy of Cyanobacteria is summarized at online databases such as AlgaeBase^19^ and CyanoDB.^20^ Discovery of a basal lineage of non-photosynthetic Cyanobacteria by phylogenetic analysis of metagenome-assembled genomes led to the proposal of expanding the phylum Cyanobacteria to include non-photosynthetic organisms (e.g. Vampirovibrionia and Candidatus Sericytochromatia).^21^^,^^22^ This drastic change has been applied to the taxonomy of databases such as SILVA^23^ but may confuse many researchers.^24^ In this study, we used the traditional definition of the phylum Cyanobacteria, which consists of only oxygenic phototrophs.

**Figure 1:**
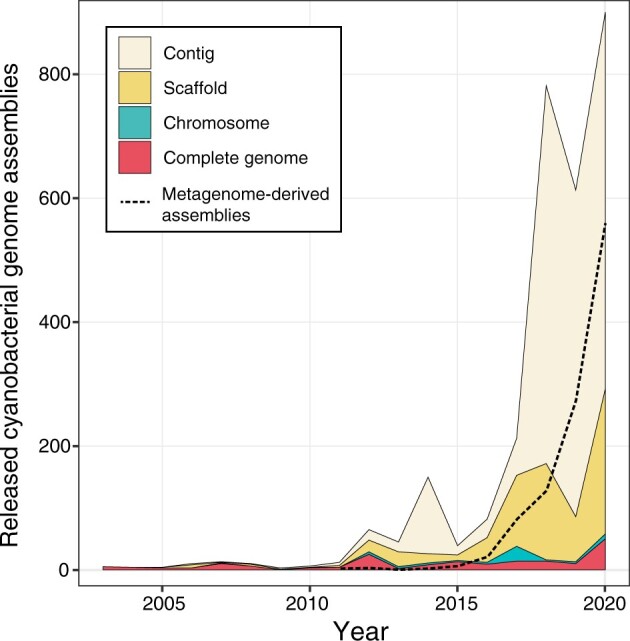
The number of genome assemblies of Cyanobacteria deposited in the International Nucleotide Sequence Database Collaboration (INSDC) databases (DDBJ, European Nucleotide Archive, and GenBank). The total number of released Cyanobacteria genomes per year is plotted. The composition of the assembly levels of each genome are shown as stacked line graphs: contig (light yellow), scaffold (light orange), chromosome (light green), and complete genome (light red). The fraction of metagenome-derived assemblies is shown as dashed line graph (black).

In 1996, the complete genome sequence of cyanobacterium *Synechocystis* sp. PCC 6803 was determined as the first genome of photosynthetic organisms.^25–27^ Approximately 25 years from this milestone, there are now 3,265 assemblies of Cyanobacteria genomes in the GenBank database (September 2021). In the National Center for Biotechnology Information (NCBI) Assembly database, the quality of each genome assembly is categorized into four levels: complete genome, chromosome, scaffold, and contig (https://www.ncbi.nlm.nih.gov/assembly/help/). The explosive increase in genome assemblies has been supported by the less contiguous genomes at the contig and scaffold levels from metagenome-derived samples (Fig. 1), in which rRNA genes (e.g. 16S and 23S rRNA) are fragmented into several repeat sequences and have often been removed from the assemblies. Genome sequencing of diverse Cyanobacteria strains deposited in the worldwide culture collections are especially important, as they are easily accessible by researchers throughout the world and have been preserved by skilled staff. Shih et al. performed genome sequencing of 54 strains of Cyanobacteria in the PCC, which include phylogenetically and phenotypically diverse strains of Sections I–V.^28^ In addition, we have been working on genome sequencing of the axenic Cyanobacteria strains in the NIES culture collection.^29–38^

In this study, we describe the genome sequencing of the 27 strains of Section IV, 2 strains from Section II, and 1 strain each from Sections III and V of the NIES microbial culture collection (total 31 strains). We mainly sequenced the heterocyst-forming strains in Section IV because their genome size is relatively large and they are expected to harbour genes with novel functions. We determined the phylogenetic relationship of the sequenced genome assemblies and also their position in the 16S rRNA phylogenetic tree including unsequenced strains. Our data will contribute to our understanding of the enormous genetic and phenotypic diversity in the phylum Cyanobacteria.

## 2. Materials and methods

### 2.1. Growth condition and DNA extraction

Cyanobacteria cells were grown in a liquid medium in the presence of a nitrogen source except for *Nodularia* sp. NIES-3585 as described in the Supplementary Table S2 and were harvested by centrifugation. Genomic DNA of the Cyanobacteria strains was purified using a NucleoBond Buffer Set III (Macherey-Nagel, Düren, Germany) and NucleoBond AXG 500 columns (Macherey-Nagel). For the strains showing low DNA yield with this method, a cell pellet of ∼100–300 mg was collected in a 2-ml tube and frozen in liquid nitrogen. The pellet was disrupted with one or two tungsten carbide beads (diameter, 3 mm; Qiagen, Hilden, Germany) using TissueLyser II (Qiagen) at 30 Hz until the cell pellet became a powder. The pellet was kept frozen by immersing the tube in liquid nitrogen during the prolonged disruption. The powdered cells were mixed with 400 µl of Tris-EDTA solution and 400 µl of Tris-buffered phenol (pH 8.0), and 1.0 g of zirconia/silica beads (diameter, 0.1 mm; BioSpec, Bartlesville, OK) at room temperature. The cells were disrupted again using the TissueLyser II at 30 Hz for 30 s and were centrifuged at 21,600*g* for 10 min at 20 °C. Approximately 400 µl of the upper (water) phase was collected, and 4 µl of RNase A (100 mg/ml) was added and incubated for 15 min at 37 °C. Next, 130 µl of Qiagen P3 buffer was added to the solution and centrifuged at 21,600*g* for 5 min at 20 °C. The supernatant was collected and added to 1.5 volumes of Qiagen buffer AW1 and loaded onto a DNeasy Plant mini column (Qiagen). Column wash and DNA elution were performed according to the manufacturer’s instructions for the DNeasy Plant mini kit (Qiagen). The eluted DNA was further purified using AMPure XP (Beckman Coulter, Brea, CA, USA) and quantified using the Qubit BR assay kit (Thermo Fisher Scientific, Waltham, MA, USA).

### 2.2. Genome sequencing, assembly, and gap-closing

Whole-genome sequencing, assembly, and gap-closing were performed using the MiSeq platform (Illumina, San Diego, CA, USA) and in silico finishing software as reported.^29^^,^^30^^,^^32–34^ For the preparation of paired-end libraries, genomic DNA was fragmented to 500–600 bp with a Covaris M-220 focused-ultrasonicator (Covaris, Woburn, MA, USA). Paired-end libraries were prepared using the KAPA HyperPrep Kit (Roche, Basel, Switzerland) with PCR-free protocol and prolonged adapter ligation for 2–3 h. Mate pair libraries with ∼8-kb insert size were prepared using the Nextera Mate pair Sample Preparation kit (Illumina) with the gel-plus protocol. Each 300-bp end of the libraries was sequenced on the MiSeq instrument with the MiSeq Reagent kit v3 (600 cycles; Illumina). Base-calling and demultiplexing of the reads were performed using Real-Time Analysis v1.18.54 (Illumina) and MiSeq Control Software v2.6.21 (Illumina). Raw sequence reads used for the assembly were deposited in the DNA Data Bank of Japan (DDBJ) Sequence Read Archive under accession numbers DRA012644 and DRA012713.

Correction of sequence errors based on the 17-mer frequency and removal of junction sequences of the mate-pair reads were performed using ShortReadManager version 0.982.^39^ Distribution of a single major peak for the 17-mer frequency was confirmed during this process to check for any apparent contamination by DNA from other organisms. Illumina adaptor sequences were removed using Cutadapt version 1.18.^40^ Processed paired-end reads and mate-pair reads were assembled using Newbler version 2.9 (Roche) with -mi 95 (minimum overlap identify 95%), -ml 70 (minimum overlap length 70 bp), -ace (produce ace file), and -a 0 (report all contigs of any length) options. We compared several assemblies with different parameters of -ml 60–100 and different input reads (30- to 150-fold coverage). The assemblies yielding the largest scaffold and lowest number of contigs (>2 kb) were subjected to *in silico* gap-closing using GenoFinisher version 2.2 and AceFileViewer version 1.6.^39^ In the Newbler output, gap sequences were produced by (i) the absence of the raw sequence read (referred to as a true gap) and (ii) the presence of repeat contigs (referred to as a false gap). The formation of a true gap can mostly be avoided by using PCR-free library preparation, whereas the sequence of a false gap can be determined *in silico* by unravelling the connections between repeat contigs. GenoFinisher provided visualization and support for the manual determination of the connection of all contigs based on the contig connection and read pair information. AceFileViewer determined the sequence variants in the repeat contigs using the read pair information. Manuals for this software are available on the website of co-author Dr Yoshiyuki Ohtsubo (http://www.ige.tohoku.ac.jp/joho/gmProject/gmhome.html). The assembly was validated using the mapping of the paired-end library and mate–pair library using FinishChecker, an accessory tool of GenoFinisher. Gene prediction and annotation were performed with the Dfast pipeline with reference to annotations from CyanoBase.^41^^,^^42^

### 2.3. Genome analysis

The year of release for the 3,265 total assemblies in the International Nucleotide Sequence Database Collaboration databases (DDBJ, European Nucleotide Archive, and GenBank) and their assembly levels (complete, chromosome, scaffolds, and contigs) were retrieved from the assembly report file of the NCBI Assembly database on 24 September 2021.^43^ Records from 2003 to 2020 were summarized using custom Perl script and visualized with ggplot2 version 3.3.0 package in R version 3.6.1 (https://www.R-project.org/). For the assignment of the taxonomy of the scaffolds, translated coding sequences (CDSs) of 31 NIES strains were downloaded from the GenBank database.^44^ Local alignment searches of the query sequences against the UniRef90 database^45^ were performed using MMseqs2^46^ with the option -s 4. The taxonomy of the subject sequence of the hit was assigned using taxonkit^47^ and custom Perl script. The top-hit (self-hit) sequence and sequences not assigned at the kingdom level (e.g. metagenome-derived cluster with TaxID = 1 in the fasta header) were removed from the hits. If two or three subjects of the top three hits were classified as phylum Cyanobacteria, the query CDS was assigned as “Cyanobacteria.” If zero or one subject of the top three hits was classified as phylum Cyanobacteria, the query CDS was assigned as “non-Cyanobacteria.” If there were fewer than two hits in total, the query sequence was assigned as “unique.” The fraction of Cyanobacterial CDSs in each scaffold was counted using custom Perl script and was visualized with the ggplot2 package in R. For the construction of the genome tree, genome assemblies of 876 Cyanobacteria strains in RefSeq database were downloaded on 16 October 2020.^48^ We removed 23 Cyanobacteria assemblies in RefSeq that showed contamination levels of >5% using checkM pipeline (Supplementary Table S4).^49^ For the genome tree construction, the search for conserved marker genes and their alignment and trimming were performed using PhyloPhlAn version 3.0.58.^50^ A phylogenetic tree of the concatenated sequence of the marker genes was constructed with RAxML version 8.2.12^51^ with PROTCATLG and a rapid bootstrap test of 100 replicates and was visualized using iTol version 4.^52^

### 2.4. Phylogenetic analysis based on 16S rRNA genes

A total of 48,174 sequences of the 16S rRNA gene from class Oxyphotobacteria (the name of this class was changed to Cyanobacteria in 2021) were downloaded from SILVA NR Ref database version 138 on 31 March 2021.^23^ Sequences of the 16S rRNA gene from 922 assemblies of RefSeq Cyanobacteria were downloaded from the NCBI website on 31 March 2021. A total of 806 sequences were chosen as representative sequences using custom Perl script and the following criteria: each chosen sequence was >1 kb and was the longest 16S rRNA sequence in each RefSeq assembly, and each chosen sequence contained mixed bases (e.g. R and N) of <20 nucleotides. Taxonomy of the RefSeq Cyanobacteria strains was assigned using the classifier of QIIME2 version 2020.8 trained with the SILVA NR Ref database.^53^ A total of 48,980 sequences of Cyanobacteria from the SILVA and RefSeq databases were combined, and sequences assigned as Chloroplast at the order level (D_3) were removed using custom Perl script, which resulted in 23,754 sequences. The remaining sequences were clustered based on a cut-off of 97% sequence identity using VSEARCH version 2.16.0 with the default parameters,^54^ which yielded 1,782 representative sequences. The clustered 1,782 sequences were further clustered based on a cut-off of 90% identity and the resultant 65 singleton sequences were removed from the input sequences, which yielded 1,717 sequences. For the outgroup, a total of 494 sequences of Vampirovibrionia were downloaded from the SILVA NR Ref database version 138 on 26 April 2021. The sequences were clustered at 97% identity using VSEARCH and the singletons were removed, which yielded 81 sequences. The 1,717 sequences of Cyanobacteria and the 81 sequences of Vampirovibrionia were aligned using SINA version 1.7.2.^55^ Sequences that were poorly aligned or contained large gaps were manually removed using AliView version 1.27.^56^ A phylogenetic tree was estimated from the aligned 1,790 sequences using IQ-TREE 2 version 2.1.2.^57^ The substitution model of TVMe+R10 was chosen as a best-fit model by ModelFinder based on the Bayesian Information Criterion.^58^ Stability of the branches in the phylogenetic tree was tested by Ultrafast bootstrap approximation with 1,000 replicates.^59^

## 3. Results and discussion

### 3.1. Genome sequencing of the NIES cyanobacteria collection

We performed genome sequencing of 31 Cyanobacteria strains in the NIES culture collection with a strategy that was established previously.^29^^,^^30^^,^^32–34^^,^^39^ Briefly, paired-end and mate-pair libraries were sequenced on the MiSeq platform, and the obtained sequence reads were assembled using Newbler and subsequent *in silico* gap closing using AceFileViewer and GenoFinisher.^39^ This approach enabled us to determine complete and nearly complete genome sequences using only Illumina short reads and substantially reduced the effort needed for Sanger sequencing to resolve the sequence gaps, which numbered in the hundreds in our assembly (Table 1). We sequenced genomes of 27 of the Section IV strains, which included diverse genera such as *Anabaena*, *Anabaenopsis*, *Aulosira*, *Calothrix*, *Cylindrospermum*, *Dolichospermum*, *Microchaete*, *Nodularia*, *Nostoc*, *Raphidiopsis*, *Scytonema*, *Sphaerospermopsis*, *Tolypothrix*, and *Trichormus* (Table 1). To improve the comprehensiveness of the genomic information of NIES strains and to demonstrate the effectiveness of our approach, strains from other sections were also sequenced: *Pleurocapsa* (Section II), *Stanieria* (Section II), *Leptolyngbya* (Section III), and *Fischerella* (Section V; Table 1).

**Table 1:** Assembly statistics of the 31 strains of the NIES collection.

Organism	Equivalent strain	Section	GenBank accession number	RefSeq ID	Total genome size (Mbp)	Chromosome size (Mbp)	Fold sequence coverage	Contings (>500 bp) before gap- closing	Scaffolds (>1 bp) in final assembly	Gaps in final assembly	16S/23S rRNA	Assembly level	CheckM completeness (%)	CheckM contamination level (%)
*Anabaena cylindrica* NIES-19	PCC 7122	IV	GCA_002367955.1	GCF_002367955.1	7.03	6.35	36	210	6	12	Assembled	Chromosome	99.46	0
*Anabaenopsis circularis* NIES-21		IV	GCA_002367975.1	GCF_002367975.1	7.07	6.57	43	284	4	12	Assembled	Chromosome	99.46	0
*Aulosira laxa* NIES-50		IV	GCA_002368055.1	GCF_002368055.1	9.35	8.46	52	265	6	11	Assembled	Chromosome	99.18	0.54
*Calothrix brevissima* NIES-22		IV	GCA_002367995.1	GCF_002367995.1	9.26	8.53	90	219	9	6	Assembled	Chromosome	99.18	0.14
*Calothrix parasitica* NIES-267		IV	GCA_002368095.1	GCF_002368095.1	9.49	8.95	88	226	6	22	Assembled	Chromosome	99.64	1.13
*Calothrix* sp. NIES-2098		IV	GCA_002368175.1	GCF_002368175.1	8.88	8.67	93	266	2	0	Assembled	Complete	99.18	0.68
*Calothrix* sp. NIES-2100		IV	GCA_002368195.1	GCF_002368195.1	9.96	9.91	65	176	2	7	Assembled	Chromosome	99.46	0.14
*Calothrix* sp. NIES-3974		IV	GCA_002368395.1	GCF_002368395.1	5.99	5.99	109	124	5	0	Assembled	Complete	100	0
*Calothrix* sp. NIES-4071		IV	GCA_002368455.1	GCF_002368455.1	12.05	11.06	57	130	9	0	Assembled	Complete	98.78	1.13
*Calothrix* sp. NIES-4101		IV	GCA_004296455.1	Not included	7.59	7.24	53	295	7	0	Assembled	Complete	99.46	1.22
*Cylindrospermum* sp. NIES-4074		IV	GCA_003994795.1	Not included	7.70	6.83	146	246	4	10	Assembled	Chromosome	99.73	0.72
*Dolichospermum compactum* NIES-806		IV	GCA_002368115.1	GCF_002368115.1	5.17	5.17	52	282	1	88	Assembled	Chromosome	99.73	0.27
*Fischerella* sp. NIES-4106		V	GCA_002368315.1	GCF_002368315.1	7.25	6.18	102	239	9	5	Assembled	Chromosome	99.46	2.04
*Leptolyngbya boryana* NIES-2135		III	GCA_002368255.1	GCF_002368255.1	7.23	6.26	59	134	4	0	Assembled	Complete	99.23	0.54
*Microchaete diplosiphon* NIES-3275		IV	GCA_002368275.1	GCF_002368275.1	10.13	–	76	402	15	74	Assembled	Scaffold	99.18	0
*Nodularia* sp. NIES-3585		IV	GCA_002218065.1	GCF_002218065.1	5.77	–	88	155	4	16	Assembled	Scaffold	99.64	0.27
*Nostoc carneum* NIES-2107		IV	GCA_002368155.1	GCF_002368155.1	9.36	8.35	59	286	4	12	Assembled	Chromosome	99.18	0.09
*Nostoc commune* NIES-4070	NEIS-2114, HK-02	IV	GCA_003990685.1	Not included	8.23	7.15	47	348	9	34	Assembled	Chromosome	99.46	0
*Nostoc linckia* NIES-25		IV	GCA_002368035.1	GCF_002368035.1	8.59	6.45	57	137	5	16	Assembled	Chromosome	100	0.82
*Nostoc* sp. NIES-2109	HK-01	IV	GCA_003990705.1	Not included	7.53	6.49	80	190	8	0	Assembled	Complete	99.46	0
*Nostoc* sp. NIES-2111		IV	GCA_002368215.1	GCF_002368215.1	7.88	6.52	83	183	9	1	Assembled	Chromosome	99.14	0.14
*Nostoc* sp. NIES-4103		IV	GCA_002368335.1	GCF_002368335.1	8.59	8.42	61	267	2	48	Assembled	Chromosome	99.09	0.54
*Pleurocapsa* sp. NIES-4102		II	GCA_002368355.1	GCF_002368355.1	5.00	4.52	85	75	7	0	Assembled	Complete	99.37	0.54
*Raphidiopsis curvata* NIES-932		IV	GCA_002368135.1	GCF_002368135.1	3.43	3.43	92	217	1	30	Assembled	Chromosome	100	0.27
*Scytonema* sp. NIES-2130	HK-05	IV	GCA_002368235.1	GCF_002368235.1	9.76	8.82	33	298	8	36	Assembled	Chromosome	99.64	4.03
*Scytonema* sp. NIES-4073		IV	GCA_002368435.1	GCF_002368435.1	9.85	9.49	73	294	6	21	Assembled	Chromosome	99.86	4.21
*Sphaerospermopsis kisseleviana* NIES-73		IV	GCA_002368075.1	GCF_002368075.1	5.61	5.35	61	394	2	153	Assembled	Chromosome	99.18	0.27
*Stanieria* sp. NIES-3757		II	GCA_002355455.1	GCF_002355455.1	5.46	5.32	50	163	2	0	Assembled	Complete	99.46	0.54
*Tolypothrix* sp. NIES-4075		IV	GCA_002218085.1	GCF_002218085.1	8.25	–	82	303	67	27	Assembled	Scaffold	100	2.67
*Tolypothrix* tenuis NIES-37	PCC 7101	IV	GCA_002368295.1	GCF_002368295.1	9.35	8.70	55	138	6	19	Assembled	Complete	99.18	0.54
*Trichormus variabilis* NIES-23		IV	GCA_002368015.1	GCF_002368015.1	7.47	6.41	97	228	6	26	Assembled	Chromosome	99.18	0

We succeeded in determining highly continuous genome sequences for a total of 31 strains from the NIES culture collection, with 9 assemblies at the complete genome level, 19 assemblies at the chromosome level, and 3 assemblies at the scaffold level (Table 1). Our high-quality continuous assemblies with a reduced number of gaps enable applications based on genome-level ultrastructure such as chromosome replication^60^ and gene synteny analyses.^61^^,^^62^ In addition, the full-length 16S and 23S rRNA genes are available in our assemblies (Table 1), which provides an association between the genome content of a given strain and a marker rRNA gene. This is important in ecological studies for predicting relevant phenotypes and genotypes based on the sequences of rRNA genes (e.g. amplicon sequencing of 16S rRNA genes). Of the nine complete genome assemblies, four are non-Section IV strains, suggesting difficulty in closing the gap for Section IV strains. The continuity of the scaffold was low only for *Tolypothrix* sp. NIES-4075, as the number of mate-pair reads was not sufficient for this strain.

The quality of our genome assemblies was estimated based on the composition of the single-copy marker genes using the checkM pipeline.^49^ The genome assemblies showed 98–100% completeness (Table 1), indicating that the genome data contained most of the conserved marker genes. The genome assemblies showed 0–4.21% contamination values (Table 1), with *Scytonema* sp. NIES-2130 (4.03%) and NIES-4073 (4.21%) showed relatively higher values. To estimate the contamination level by a different approach, the CDSs in each scaffold were searched using MMSeqs2 against UniRef90, a database that was built by clustering UniRef sequences with ≥90% identity.^45^ This analysis revealed that the majority of the CDSs in the scaffolds originated from Cyanobacteria (Supplementary Fig. S1), suggesting that our genome assemblies do not contain heterogeneous scaffolds derived from other phyla.

### 3.2. Phylogenetic position of the sequenced strains

**Figure 2:**
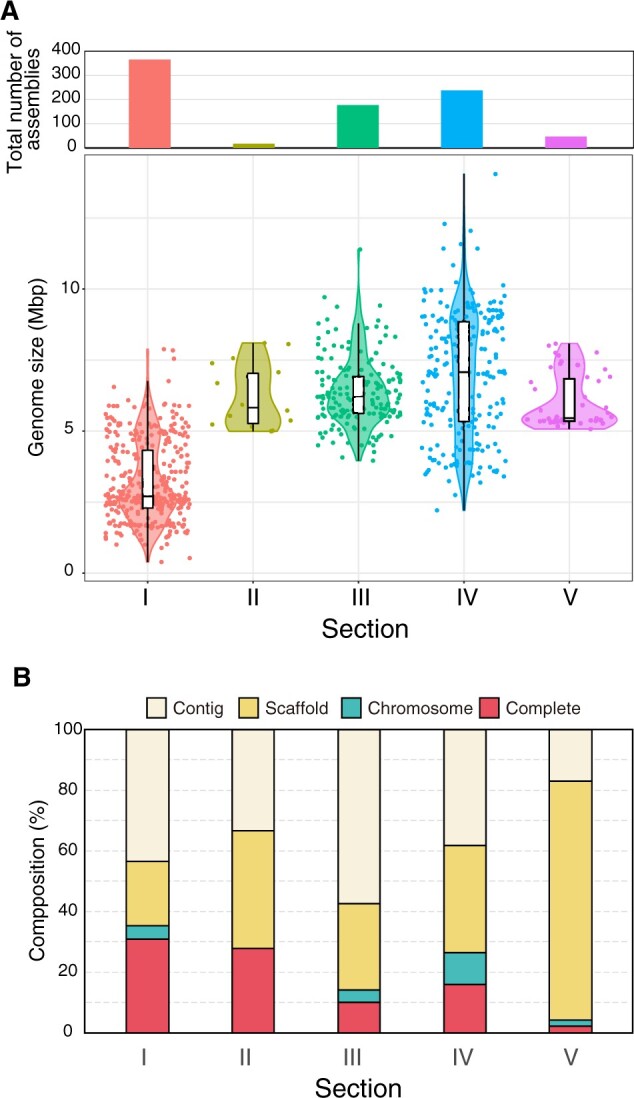
The number of Cyanobacteria genome assemblies in the RefSeq database. (A) The total number of assemblies (upper) and distribution of the genome size (lower) are plotted for each Cyanobacteria section. The box plots show first and third quartiles. The middle line in the box shows median genome size. The upper and lower whiskers show maximum and minimum genome size, which extended no more than 1.5-folds of distance between the first and third quartiles. The violin plots show the distribution of genome size of each assembly. (B) The composition of the assembly levels from this study was plotted for each Cyanobacteria section. We did not include assemblies with unassigned sections.

The RefSeq database provides a comprehensive, integrated, non-redundant, and well-annotated set of genome sequences.^48^ We summarize here the morphological classification, assembly level, and genome size of 855 assemblies from Cyanobacteria in the RefSeq database including the sequenced NIES strains (Fig. 2, Supplementary Fig. S2 and Supplementary Table S3). The total number of genome assemblies for Cyanobacteria in RefSeq was high for Section I (366 assemblies), Section IV (238), and Section III (178) as compared with Section V (47) and Section II (18). Distribution of genome size among Section I members showed two peaks: the one at ∼2.5 Mb contained oceanic strains such as *Synechococcus* and *Prochlorococcus*, and the other at ∼5 Mb contained freshwater strains such as *Microcystis* (Fig. 2A and Supplementary Table S3). Notably, the median genome size was largest (∼7.08 Mb) in Section IV with substantial variation from a minimum of 2.21 Mb for the symbiotic cyanobacterium *Richelia intracellularis* to a maximum of 14.1 Mb for *Scytonema* sp. (Fig. 2A and Supplementary Table S3). We deposited 4 assemblies at the complete genome level, 16 assemblies at the chromosome level, and 3 assemblies at the scaffold level for Section IV strains into RefSeq (Table 1). Thus, the number of our sequenced strains corresponds to ∼10% of Section IV strains with sequence information in RefSeq, and thus they contribute to the development of a high-quality genome database (Fig. 2B).

**Figure 3:**
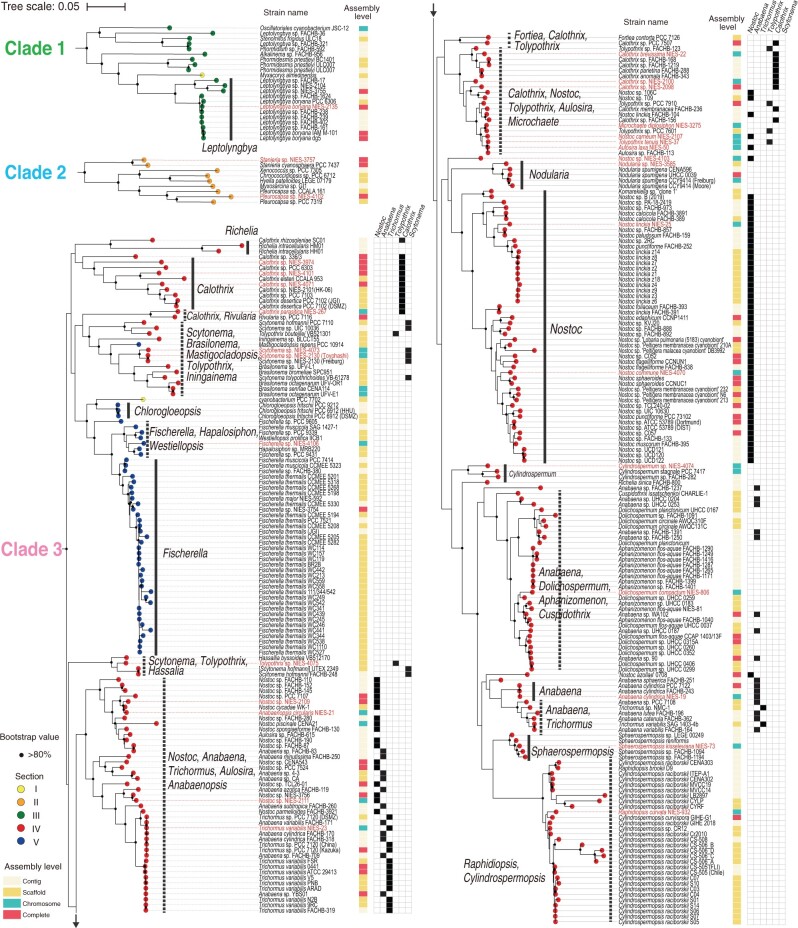
The phylogenetic position of the sequenced NIES strains from this study in the RefSeq database. The phylogenetic relationships among the 31 sequenced strains from the NIES collection are shown within the context of all 855 Cyanobacteria genomes in the RefSeq database. The phylogenetic tree was estimated with the ML method based on the conserved marker genes using PhyloPhlAn (see Supplementary Fig. S2 for the entire tree). Clades 1, 2, and 3, which contain the 31 sequenced NIES strains from this study (red), are enlarged here. The morphological classifications of each strain are shown as coloured circles at the tips of the phylogenetic tree: Sections of I (yellow), II (orange), III (green), IV (red), V (blue), and unassigned (purple). The assembly levels of each genome are shown as coloured stripes: contig (light yellow), scaffold (light orange), chromosome (light green), and complete genome (light red). Major subclades made up of a single genus (solid black lines) and multiple genera (dashed black lines) are shown. The distribution of members of the genera *Nostoc*, *Anabaena*, *Trichormus*, *Tolypothrix*, *Calothrix*, and *Scytonema* are shown (filled black squares). Bootstrap values of >80% in the 100 replications are shown with a black circle.

High-quality genome sequences enable us to estimate the phylogenetic tree of host organisms based on multiple sequence alignment of the dozens to hundreds of concatenated marker genes. This approach yields a more accurate phylogenetic tree as compared with the approach of using a single marker gene (e.g. 16S rRNA gene). RefSeq contains some contaminated assemblies even with the curation process.^63^ We thus applied the checkM pipeline to the 876 Cyanobacteria strains in RefSeq and removed 23 strains with a threshold contamination level of >5% (Supplementary Table S4). Assemblies from 31 strains in the NIES collection and 824 additional strains in RefSeq were subjected to the PhyloPhlAn pipeline to estimate the maximum likelihood (ML) tree based on the concatenated marker genes.^50^ Sequenced NIES strains were found in three clades in the constructed phylogenetic tree, which were designated as Clades 1, 2, and 3 (Supplementary Fig. S2). In Clade 1 of Section III, *Leptolyngbya boryana* NIES-2135 was closely related to *Leptolyngbya boryana* PCC 6306 but was more distantly related to *Leptolyngbya* sp. NIES-2104 and NIES-3755 (Fig. 3). In Clade 2 of Section II, *Stanieria* sp. NIES-3757 was related to *Stanieria cyanosphaera* PCC 7437, whereas *Pleurocapsa* sp. NIES-4102 was but distantly related to *Pleurocapsa* sp. PCC 7319 (Fig. 3). The 28 heterocystous NIES strains were distributed to Clade 3 with a wide variety of phylogenetic locations (Fig. 3). *Calothrix* NIES-3974 was found to be a distinctly deep branched strain. *Calothrix* NIES-267 forms a monophyletic lineage with *Rivularia* sp. PCC 7116 and appeared to be in a deep-branched lineage. Such deeply branched strains may provide new clues as to how heterocystous Cyanobacteria evolved.

In Clade 3, several major subclades are composed of a single genus (e.g. *Fischerella*, *Nostoc*, *Nodularia*, *Sphaerospermopsis*, *Chlorogloeopsis*, and *Cylindrospermum*) (Fig. 3, thick solid lines). However, we also found subclades that consist of heterogeneous genera (Fig. 3, thick dashed lines). Here we focus on those genera that are considered most problematic from a taxonomic perspective: *Nostoc*, *Anabaena*, *Trichormus*, *Tolypothrix*, *Calothrix*, and *Scytonema*. *Nostoc*, *Anabaena*, and *Trichormus* share similar morphological characteristics: they are uniseriate and isopolar and have metameric trichomes. *Nostoc* can form small motile filamentous cells called hormogonia, whereas *Anabaena* and *Trichormus* do not. *Anabaena* and *Trichormus* are distinguished by the relative position of their akinetes, dormant cells that form under stress conditions and have thick cell walls. Anabaena forms akinetes right next to or 2-4 cells away form the heterocyst (paraheterocytic), whereas *Trichormus* forms them far from the heterocyst (apoheterocytic). *Tolypothrix* has mature trichomes that show a low degree of tapering with an apical–basal polarity, whereas *Calothrix* trichomes show a high degree of tapering.^64^*Scytonema* has trichomes that do not show apical–basal polarity. *Tolypothrix* and *Scytonema* form false-branched filaments. The heterologous distribution of these genera on our genome tree may indicate the plasticity of these morphological characteristics, which is problematic for taxonomic assignment of these genera based on morphological observations alone. Indeed, we observed morphological differences among *Aulosira laxa* NIES-50, *Tolypothrix tenuis* NIES-37, *Nostoc carneum* NIES-2107, and *Microchaete diplosiphon* NIES-3275 by light microscopy, but more detailed analyses (e.g. observation under nitrogen-deficient conditions and electron microscopy) are necessary to validate their taxonomic assignments. We also found that *Scytonema millei* VB511283 located in the subclade of the genus *Chroococcidiopsis* of Section II (Supplementary Fig. S2), which may have resulted from an error during the sequencing process of this strain.^65^

Another example of the phenotypic plasticity of Cyanobacteria is found in the taxonomic assignment of the subclade containing *Raphidiopsis* and *Cylindrospermopsis* (Fig. 3). The two genera are uniseriate, isopolar, gradually attenuated towards both ends of the filaments and can form akinetes. Their only difference is that *Raphidiopsis* does not form heterocyst, whereas *Cylindrospermopsis* does. Recent studies showed that *Raphidiopsis* and *Cylindrospermopsis* form a monophyletic subclade with the intermixed position in the phylogenetic tree based on 16S rRNA and spacer regions.^66^ This suggests the occurrence of the loss of the capacity of heterocyst formation during the evolution of this subclade. Our genome-based phylogenetic tree agrees with this report and supports the unification of the two genera under the name *Raphidiopsis*.^66^

Walter et al. proposed a new taxonomy of Cyanobacteria based only on the robust phylogenetic tree generated from the conserved marker genes.^67^ Komarek expressed concern over this approach because of the lack of descriptions and typification.^68^ We agree that phenotypes and genotypes are tightly coupled in some cases, for example, cell pigmentation and photosynthetic genes in our previous study.^69^ However, the relationship between the morphology and gene content of Cyanobacteria has not been fully elucidated. We do not yet know which gene sets are necessary and sufficient for the ability to form hormogonia, for example. Therefore, it is difficult to reliably predict whether a given strain of Cyanobacteria has the ability to form hormogonia and therefore to assign that strain to the genus *Nostoc* or *Anabaena* based only on its whole-genome information. As this example shows, it is reasonable to use the polyphasic approach for Cyanobacteria taxonomy as proposed by Komarek et al., which combines phylogenetic data, morphological data, and ecophysiological data.^18^ The isolation and cultivation of diverse Cyanobacteria strains in worldwide culture collections are important for this approach. Researchers can order individual Cyanobacteria strains within a heterogenic clade from the culture collection and can compare their phenotype(s) in detail under controlled experimental conditions. It should be mentioned that the establishment of cryopreservation protocols for the isolated strains is necessary for their reproducible assignment, as certain phenotype(s) will be lost during cultivation.

### 3.3. Phylogenetic tree based on 16S rRNA sequences

**Figure 4:**
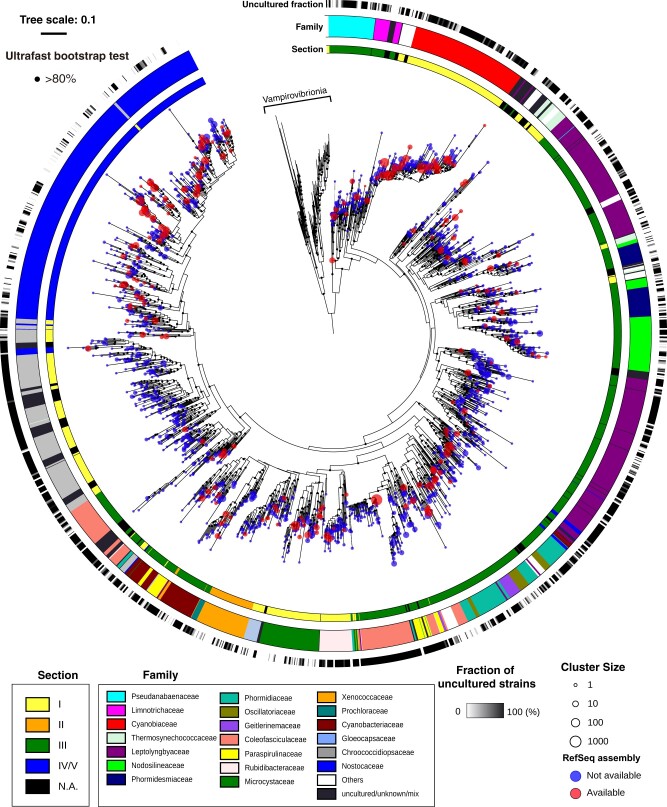
A phylogenetic tree for 1,709 strains of Cyanobacteria based on their 16S rRNA gene sequences in the SILVA database. The phylogenetic tree was estimated based on 1,709 sequences from the SILVA database for Cyanobacteria 16S rRNA genes clustered at 97% identity. Section- and family-level assignments of the sequences based on the SILVA taxonomy are shown. The number of sequences in each cluster is indicated by the circle sizes. Clusters containing RefSeq assemblies are colored in red. The fraction of uncultured strains was estimated from the taxonomic assignments in SILVA (e.g. taxonomy containing ‘Uncultured’) and is plotted on the tree (black/white gradient).

The tree generated from whole-genome data can inform the phylogenetic relationship of the sequenced strains, but the phylogenetic tree generated from 16S rRNA sequences provides more information about strains that we have not yet sequenced. We obtained all the available approximately 24,000 sequences corresponding to 16S rRNA sequences from Cyanobacteria from the SILVA Ref database and clustered them at a threshold of 97% identity. A total of 1,790 representative sequences of the clusters, which included 81 Vampirovibrionia sequences as the outgroup, were aligned and subjected to phylogenetic tree construction with the ML method (Fig. 4). We examined whether each cluster contains any RefSeq assemblies (Fig. 4, red circles). The family-level assignments of the sequences in the SILVA database, which are more robust than the fluctuating genus-level assignments, are plotted on the phylogenetic tree (Fig. 4, outer coloured stripe). We also indicated the section for each family (Fig. 4, inner coloured stripe). Moreover, we estimated the fraction of uncultured strains in each cluster and plotted them on the phylogenetic tree (Fig. 4, black stripe).

Section III is the most abundant with respect to the number of the clustered 16S rRNA sequence of Cyanobacteria (∼50%, 816 of 1,626 strains that were assigned a Section) (Fig. 4 and Supplementary Table S5). This contrasts that Sections I and IV represent the majority with sequenced genomes in the RefSeq database (Fig. 2A). This comparison highlights the importance of carrying out genome sequencing of Section III strains as the next focus for Cyanobacteria genome sequencing. The morphological diversity of Section III is smaller than that of other filamentous Sections IV and V, and this may cause the bias of the genome sequence. One of the sparsely sequenced groups of Section III is the Leptolyngbyaceae (Fig. 4, purple stripe in the family-level assignment). *Leptolyngbya* is the major genus of Leptolyngbyaceae and is characterized by a simple morphology of narrow (0.5–3 µm) cylindrical trichomes. The polyphyly of *Leptolyngbya* was shown by the phylogenetic tree of concatenated marker genes of sequenced strains (Supplementary Fig. S2) and by that of 16S rRNA gene (Fig. 4). Our previous study showed that differences in gene contents among terrestrial NIES-2104 strain and freshwater PCC 6306 strain of *Leptolyngbya* (Supplementary Fig. S2).^61^ Such genomic study will elucidate great genetic diversity among *Leptolyngbya* strains having similar morphology. There are also few sequenced genomes in the Chroococcidiopsaceae of Section I, which includes genera such as *Aliterella*, *Chroococcidiopsis*, *Dapisostemonum*, and *Myxosarcina* (Fig. 4, grey stripe in the family-level assignment). Most of the Chroococcidiopsaceae showed a high uncultured fraction, suggesting that the isolation and cultivation of these strains represent the limiting step for this family (Fig. 4, black stripe in the uncultured fraction). In contrast, the Nostocaceae of Section IV, Xenococcaceae of Section II, and Microcystaceae of Section I showed lower uncultured fractions as compared with other families.

Our genome tree suggests that the taxonomic inconsistencies in Cyanobacteria are, to some extent, due to morphological plasticity (Fig. 3). The inconsistency of the taxonomy has a negative impact on research involving Cyanobacteria. When researchers detect Cyanobacteria strains based on the amplicon sequencing of 16S rRNA, the taxonomy of these sequences can be assigned only using reference 16S rRNA sequences. For example, if the detected sequence is very similar to that of the *Nostoc* and *Anabaena*, the genus-level assignment of this sequence becomes uncertain and would be classified as Nostocaceae cyanobacterium. Thus, the heterogeneity of taxonomy of closely related strains decreases the sensitivity of taxonomic assignment with this technique. In addition, there are some inconsistencies between the phylogenetic position and the family-level assignments in the SILVA database. For example, the genus *Calothrix* clearly belongs to the family Nostocaceae but some strains of *Calothrix* are misclassified in the family Leptolyngbyaceae in SILVA (Supplementary Table S5). Furthermore, taxonomic classification system of SILVA is different from CyanoDB. For example, *Phormidesmis* strains mostly belong to Phormidesmiaceae in SILVA (Fig. 4), but they belong to Leptolyngbyaceae in CyanoDB, since Phormidesmiaceae is not validated in this database. Curation of taxonomic assignment within the reference 16S rRNA database is necessary for the accurate taxonomic assignment of Cyanobacteria based on amplicon sequencing.

## 4. Conclusion

We have determined genome sequences for 28 heterocystous and 3 non-heterocystous strains of Cyanobacteria (total 31 strains). We also determined the phylogenetic relationship of the Cyanobacteria in RefSeq and found inconsistencies in the taxonomy assignments of some genera of Section IV and their phylogenetic positions. This is most likely caused by the morphological plasticity of Cyanobacteria, which is problematic for some applications such as amplicon sequencing. Isolation, cultivation, and cryopreservation of diverse Cyanobacteria strains in worldwide culture collections will help to overcome this problem by providing the opportunity for reproducible and re-analysable taxonomic assignment. Our phylogenetic analyses also suggest that the isolation, cultivation, and genome sequencing of Section III strains are important in the future.

## Supplementary Material

dsab024_Supplementary_Data
