# Supplementary figures and images for: Genome sequencing of the NIES Cyanobacteria collection with a focus on the heterocyst-forming clade
Source: DNA Res. 2021 Oct 22;28(6):dsab024. doi: 10.1093/dnares/dsab024 (PMC8634303; doi:10.1093/dnares/dsab024)

Fig. S1

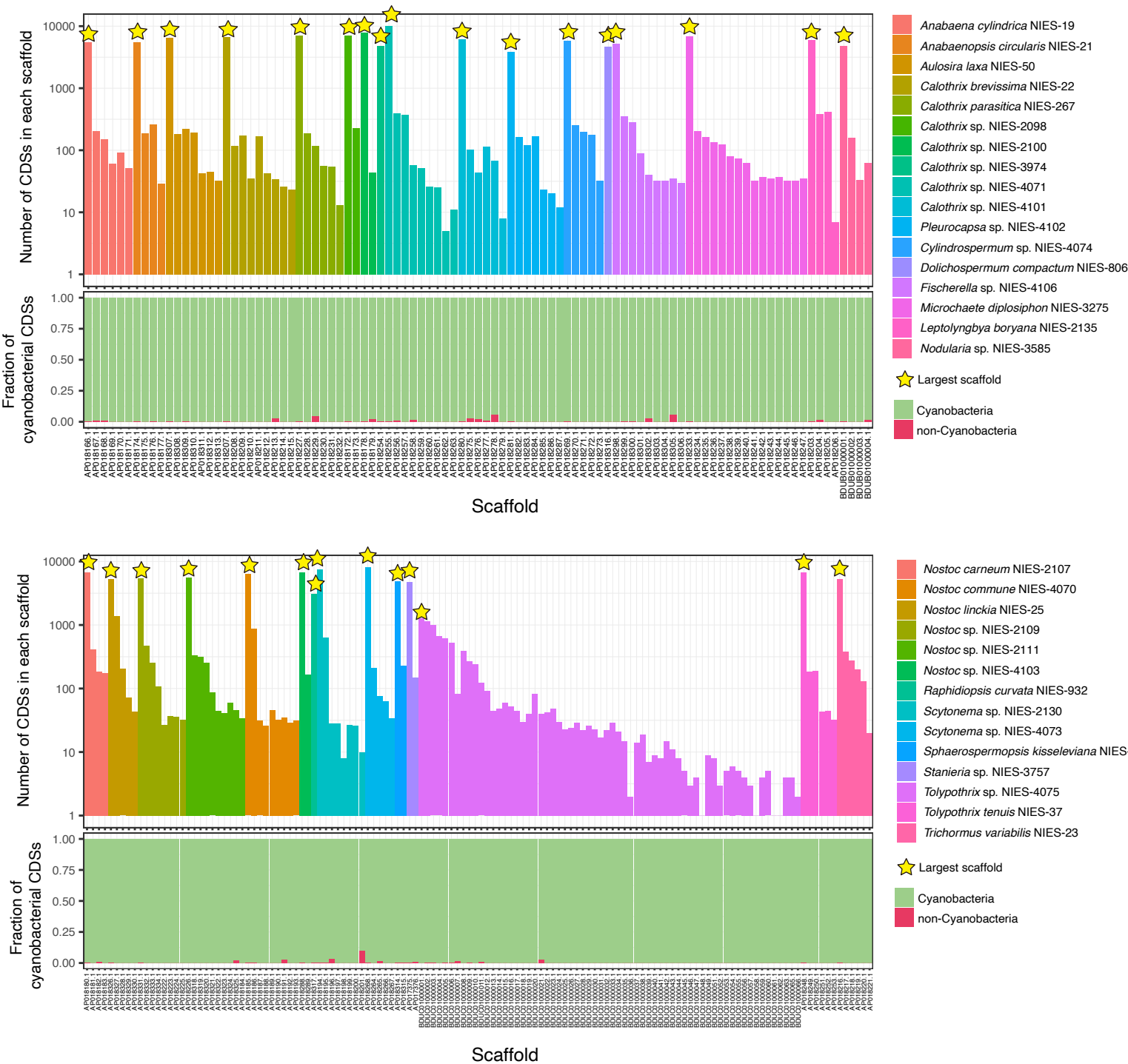

Supplement: dsab024_Supplementary_Data [file dsab024_supplementary_data.zip › Fig.S1.pdf]

Fig. S2

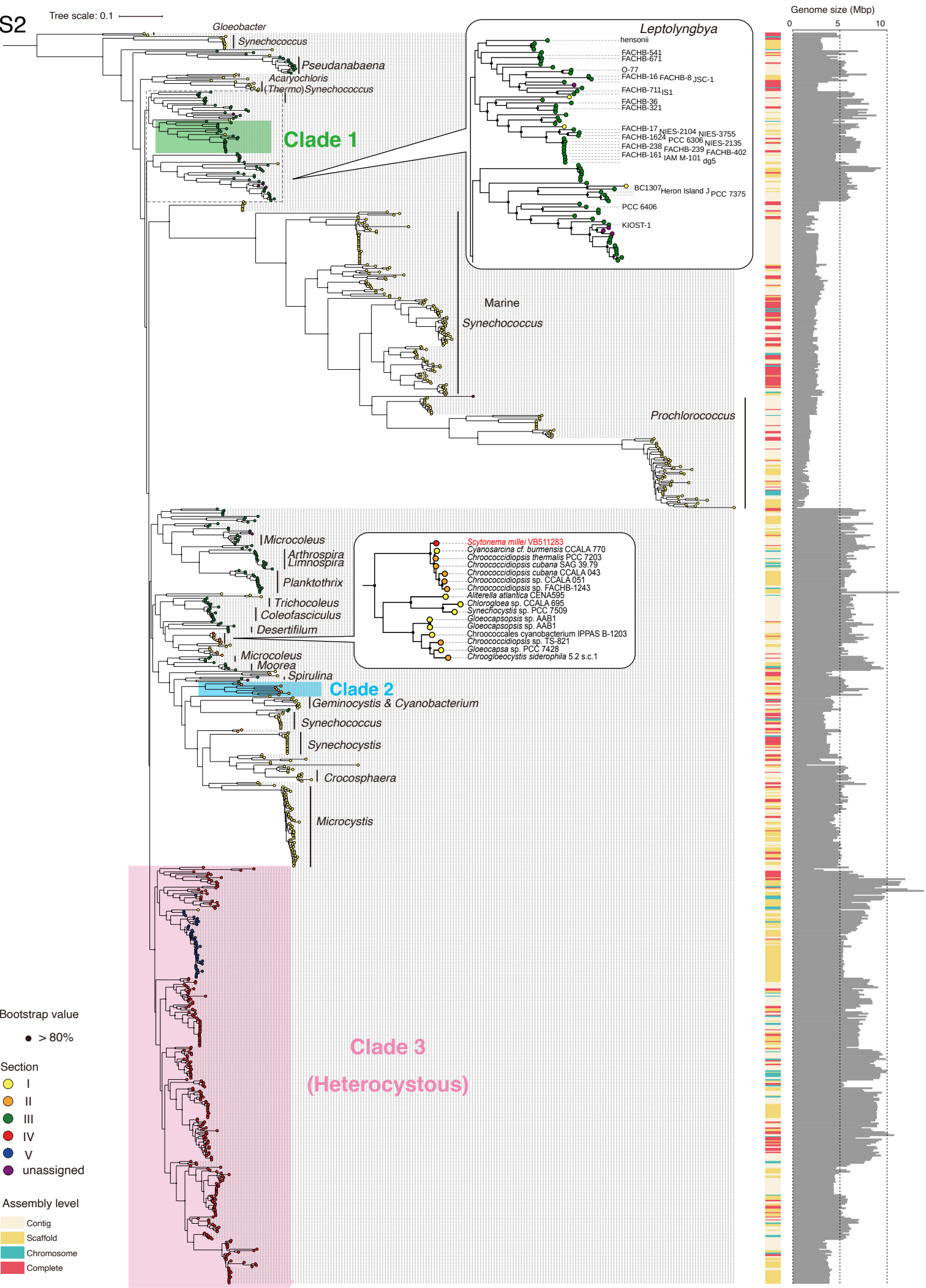

Supplement: dsab024_Supplementary_Data [file dsab024_supplementary_data.zip › Fig.S2.pdf]
